# Assessing the population coverage of a health demographic surveillance system using satellite imagery and crowd-sourcing

**DOI:** 10.1371/journal.pone.0183661

**Published:** 2017-08-31

**Authors:** Aurelio Di Pasquale, Robert S. McCann, Nicolas Maire

**Affiliations:** 1 Department of Epidemiology and Public Health, Swiss Tropical and Public Health Institute, Basel, Switzerland; 2 University of Basel, Basel, Switzerland; 3 Laboratory of Entomology, Wageningen University and Research Centre, Wageningen, The Netherlands; Institute of Microbial Technology CSIR, INDIA

## Abstract

Remotely sensed data can serve as an independent source of information about the location of residential structures in areas under demographic and health surveillance. We report on results obtained combining satellite imagery, imported from Bing, with location data routinely collected using the built-in GPS sensors of tablet computers, to assess completeness of population coverage in a Health and Demographic Surveillance System in Malawi. The Majete Malaria Project Health and Demographic Surveillance System, in Malawi, started in 2014 to support a project with the aim of studying the reduction of malaria using an integrated control approach by rolling out insecticide treated nets and improved case management supplemented with house improvement and larval source management. In order to support the monitoring of the trial a Health and Demographic Surveillance System was established in the area that surrounds the Majete Wildlife Reserve (1600 km2), using the OpenHDS data system. We compared house locations obtained using GPS recordings on mobile devices during the demographic surveillance census round with those acquired from satellite imagery. Volunteers were recruited through the crowdcrafting.org platform to identify building structures on the images, which enabled the compilation of a database with coordinates of potential residences. For every building identified on these satellite images by the volunteers (11,046 buildings identified of which 3424 (ca. 30%) were part of the censused area), we calculated the distance to the nearest house enumerated on the ground by fieldworkers during the census round of the HDSS. A random sample of buildings (85 structures) identified on satellite images without a nearby location enrolled in the census were visited by a fieldworker to determine how many were missed during the baseline census survey, if any were missed. The findings from this ground-truthing effort suggest that a high population coverage was achieved in the census survey, however the crowd-sourcing did not locate many of the inhabited structures (52.3% of the 6543 recorded during the census round). We conclude that using auxiliary data can play a useful role in quality assurance in population based health surveillance, but improved algorithms would be needed if crowd-sourced house locations are to be used as the basis of population databases.

## Introduction

Detailed, high resolution and up-to-date maps on human settlements are not available for many rural areas in low and middle income countries, but such information on human population distribution would be invaluable for measuring precisely the impacts of population growth, for monitoring changes and for planning interventions [1], in particular in the health sector. The absence of such information makes the planning and implementation of field studies of public health a challenge in these places.

One approach to resolving these challenges is to establish Health and Demographic Surveillance Systems (HDSS). An HDSS is a system that collects longitudinal data on core demographic events (births, deaths, migration, and relationships) and certain health indicators at regular intervals (normally between 3–4 times per year) from a target population in an area where government-based data for these events and indicators are unreliable due to total absence of a Civil Registration System (CVRS) in the area or improperly recorded data [2]. HDSS are an importance source of demographic information in areas where routine vital registration is absent or incomplete and serve as sampling frames for intervention trials, providing a comprehensive list of households to be selected when monitoring trial outcomes. Without an HDSS, the absence of high resolution population maps makes establishing the level of population coverage inherently difficult.

The Majete Malaria Project [3] in Malawi (MMP) is an operational research project in southern Malawi that aims to increase community participation in malaria control through education and community engagement, and to study the impact of structural house improvements and larval source management on malaria transmission when implemented in addition to standard malaria control interventions [3].

Ensuring completeness and accuracy of the population database is essential for accurate characterization of core demographic as well as key health indicators in an HDSS, but ground censuses are labor-intensive, time-consuming, and are not necessarily complete. Unlike ground-truthed maps of house locations, high-resolution satellite images are generally available [4], and easy to access through an application programming interface (*API*). There are many popular or less popular available online as Google Maps, Bing Maps, OpenStreetMaps, and MapQuest… All provide similar services with some more specialized on a specific feature (traffic, driving directions, education etc…). Bing map was used rather than Google Maps because of a suspected incompatibility between the OpenLayer library and Google Maps at the time of the development of the web application.

We present an approach for estimating the population coverage of an HDSS using geolocations of buildings, crowd-sourced from satellite imagery, to assess the completeness of the population data. This exploits features of the OpenHDS data system, which is increasingly used as a standard in HDSS sites, combined with volunteered geographic information (VGI) [5], and show the results of applying this to the area of the MMP.

A crowd-sourcing approach was used to collect geo-locations of houses in the study area of the MMP from satellite images. This was used to establish a database of building geolocations for comparison with that established from the census of the population by field teams, allowing us to identify buildings which were possibly missed in the HDSS census. The population coverage of the census for the HDSS was estimated on the basis of visits by a supervisor to a sample of locations identified as buildings on the satellite images but absent from the census database at the end of the census-round (ground-truthing).

## Methods

### Population

The HDSS is run in the Chikhwawa District, an area in the lower Shire River Valley region of southern Malawi. The district, mainly rural, has a population of over 530,000 people distributed in an area of about 4,800 km2 [6]. Since starting in 2014, MMP has initially concentrated efforts in three regions, referred to for convenience as focal areas A, B and C, respectively. Focal areas were delineated to cover the same villages as those targeted by one of MMP’s implementing partners, The Hunger Project [7], and spaced roughly evenly around Majete Wildlife Reserve (MWR) to capture a maximum amount of the ecological variation present in the area. Villages neighboring these three focal areas, but which were not covered by The Hunger Project, were not eligible to be enrolled in the HDSS.

### Data system

The HDSS data were managed using the OpenHDS System [6,8,9]. OpenHDS is an HDSS data system developed on a standard relational database management system (Mysql, Postgress, MS SQL Server etc.), designed and developed to enable simpler and more robust data collection and data management routines than possible with paper-based data collection traditionally used in population-based surveillance. Data collection in the field uses an application running on tablet computers.

The OpenHDS System [8,10] was set up at the startup of the HDSS in Majete, requiring installation of server components of the system on local server. The OpenHDS system is interfaced with the Open Data Kit (ODK) an open source suite of tools to author, manage and run data collection with mobile devices [11]. Samsung Galaxy Tab-3 Android tablets running the version 4.1.2 of Android (Jelly Bean) [12] were configured with the OpenHDS mobile [13] and the ODK Collect [14] applications, to communicate with the ODK Aggregate [11] and OpenHDS web [15] component through the wi-fi network at the field station.

During the census, demographic surveillance visit to each location in the study site, location coordinates were captured through the tablets’ in-built GPS to build a database of inhabited buildings. This approach allowed the aggregation of data points in a central database in near-time, i.e. within days after a house was visited.

### Field data collection

12 fieldworkers recruited from the target communities for their knowledge of the area and to ensure good relations with the communities were trained on the use of the OpenHDS mobile system.

**Fig 1:**
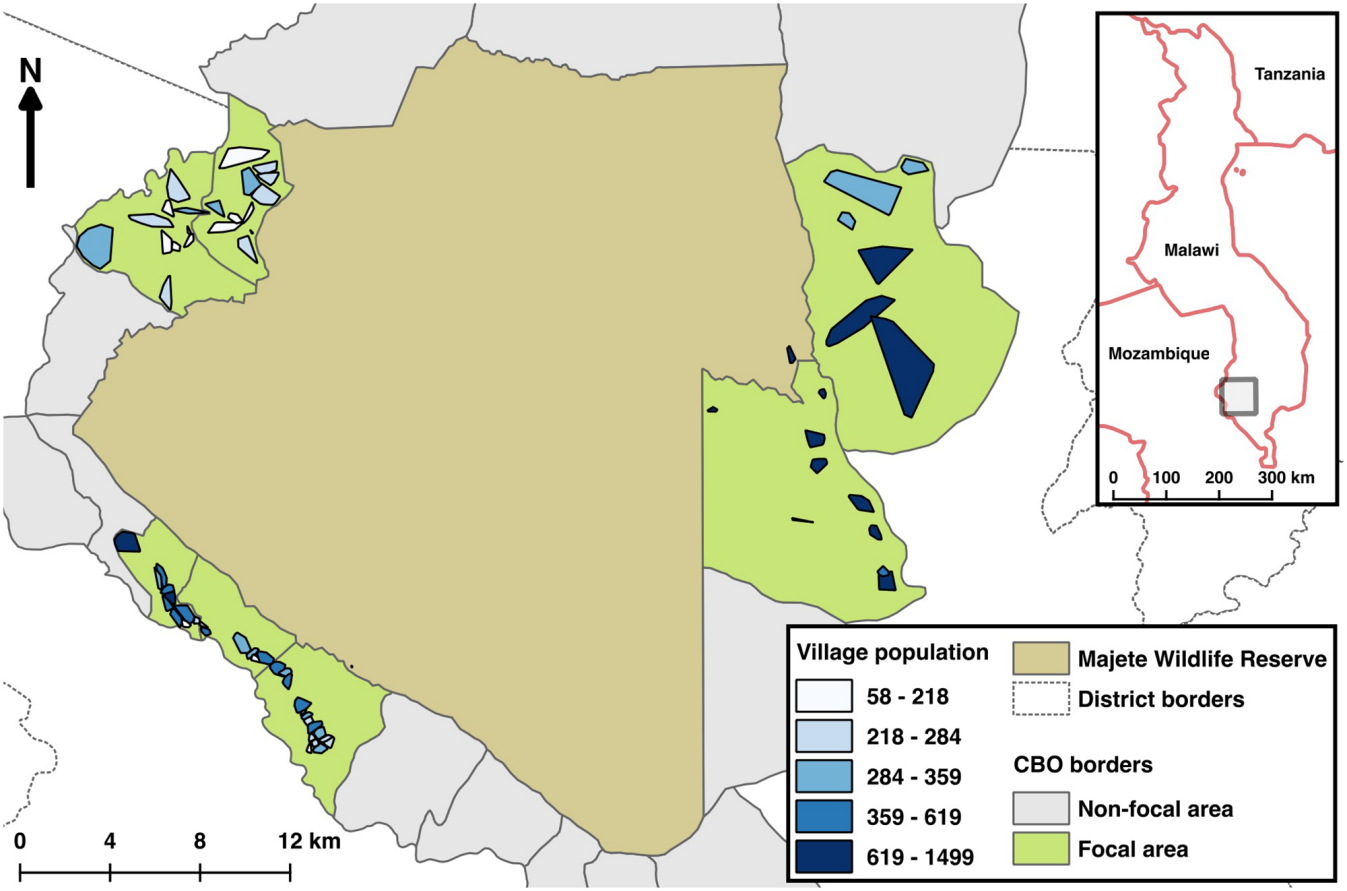
Map showing Majete Wildlife Reserve, surrounded by 19 groups of villages known as community-based organizations (CBO). The 62 villages enumerated in the current study are located in three focal areas. Village populations are as indicated in the legend (Reprinted with slight modification from Kabaghe et al 2017 under a CC BY license, with permission from PLOS, original copyright 2017).

Each HDSS has a defined location hierarchy in the area under surveillance. The lowest level of this location hierarchy is the one leading the ID generation for the HDSS entities and is important in our study for the identification of houses that became part of the ground-truthing (see section “Ground truthing”). A complete list of the villages (lowest location hierarchy level) was obtained from African Parks-Majete, the management authority of MWR and implementing partner in MMP. In the area targeted for the HDSS surrounding MWR, there are 62 villages (Fig 1).

**Fig 2:**
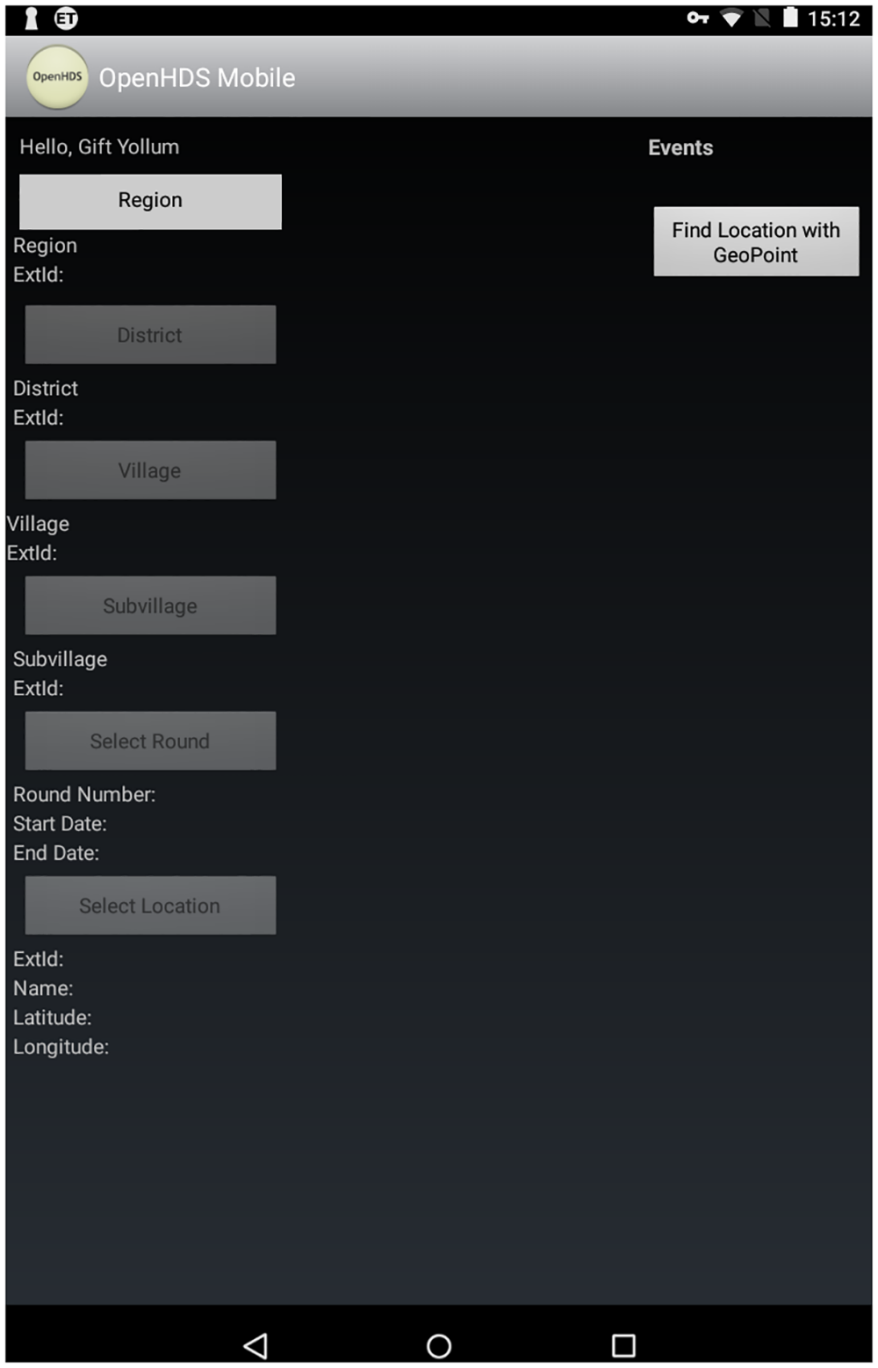
OpenHDS mobile application snapshot of location hierarchy selection.

At village level during the census round, the fieldworker collected location information where individuals were living. This task was performed through OpenHDS mobile integrated with the ODK collect application. After the login into the OpenHDS mobile application, the fieldworker had to select the village where the house was located, going through selecting the hierarchies available in the OpenHDS mobile (Fig 2). Once he selected the village, he had to create the location by pressing the create location button. An ODK Xform was automatically opened on the tablet, pre-filled with data previously selected in the OpenHDS app, plus a unique ID identifying the fieldworker, a unique ID associated to the location automatically generated through the OpenHDS mobile application according with the INDEPTH standardized identifiers [4,16], and the date of the visit to the location. At this point, the coordinates of the location were recorded by the fieldworker using the GPS sensor of the tablet. If the sensor reported accuracy of 5 meters or less, the coordinates were recorded automatically. In cases where such accuracy could not be reached due to weak GPS signal, the fieldworker was allowed to manually accept a positioning with lesser accuracy. The information of the locations was then transferred to the system central database.

### Volunteered locations

**Fig 3:**
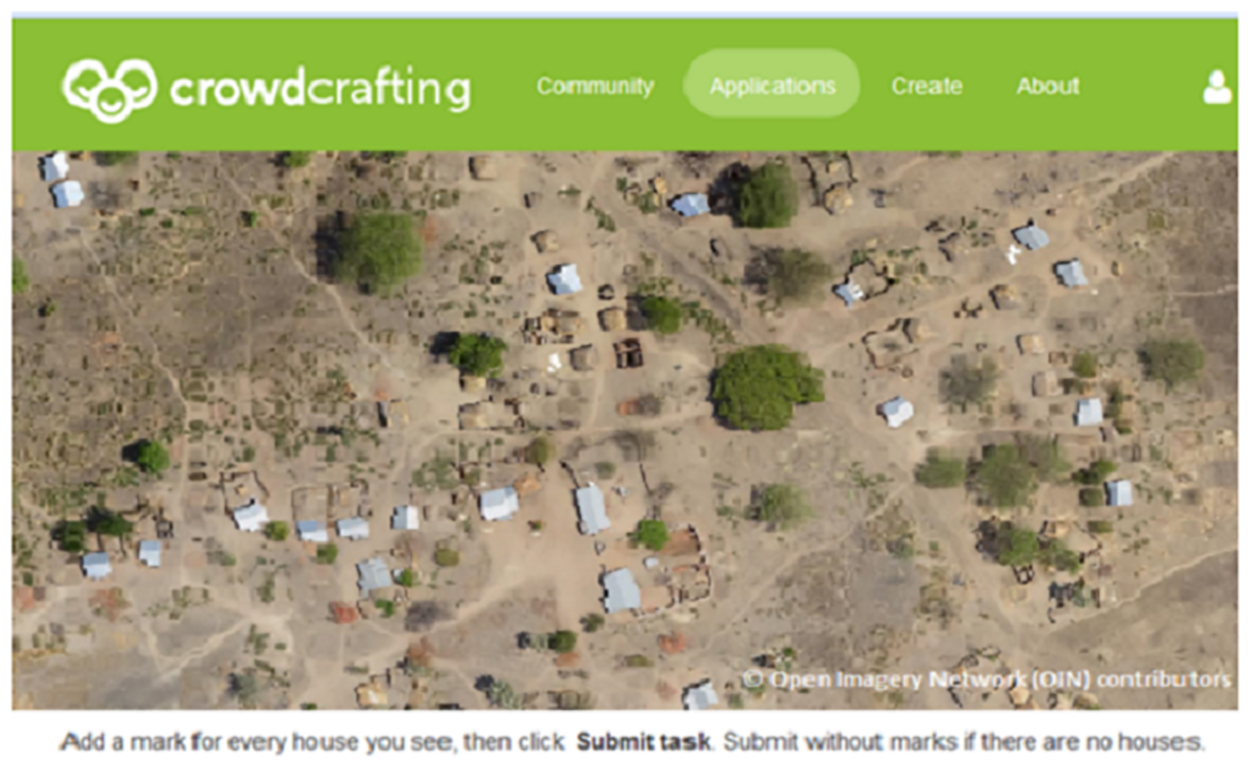
Rural Geolocator: A web-application for identifying houses on satellite images by visual inspection (illustrative purposes only).

A software application called Rural Geolocator was developed using the PyBossa framework [17] and the Openlayers library [18] to present satellite images from Bing [19–21], of the study areas in a web-browser [22]. PyBossa (inspired by the Bossa platform [23]), is an open-source platform for applications using human interaction or recognition through the help of volunteers (crowd-sourcing) to obtain information that a machine alone cannot easily deduce. Rural Geolocator was hosted on the easily accessible crowd-sourcing platform named crowdcrafting.org [24–26] (Fig 3). Volunteers were recruited via this platform by advertising the project on crowdcrafting.org, and on social media. The volunteers were provided with a simple and well defined task each time, which consisted of visually inspecting a small section of the study area (300-350m x 500-600m) and marking all potentially inhabitable structures using mouse clicks. If no houses were spotted in the determined area, the volunteer would submit the task without marking anything. Tasks were replicated at least three times, i.e. each section was processed by a minimum of three different volunteers. Volunteers were distinguished either by their user id (for registered volunteers), or on the basis of the IP address of their computer (for anonymous volunteers). Replicate results submitted for each task were consolidated using the following clustering approach: contributed geolocations were processed sequentially and added to a set of points, but only if the set did not yet contain a location less than 10m away. In case such a location was already in the set, it was replaced with a location mid-way between the contained and currently processed point. The number of replicates contributing to each of the geolocations in the resulting dataset was recorded.

The tasks processed by the volunteers were grouped into three batches corresponding to the three focal areas described above (A, B, and C). The batch sizes for focal areas A, B and C were and 682, 2953, and 1031 tasks, respectively. The area covered by each batch was defined prior to the completion of the HDSS census, and therefore the spatial extent of each batch was greater than the village borders eventually identified by the HDSS census.

### Ground truthing

**Fig 4:**
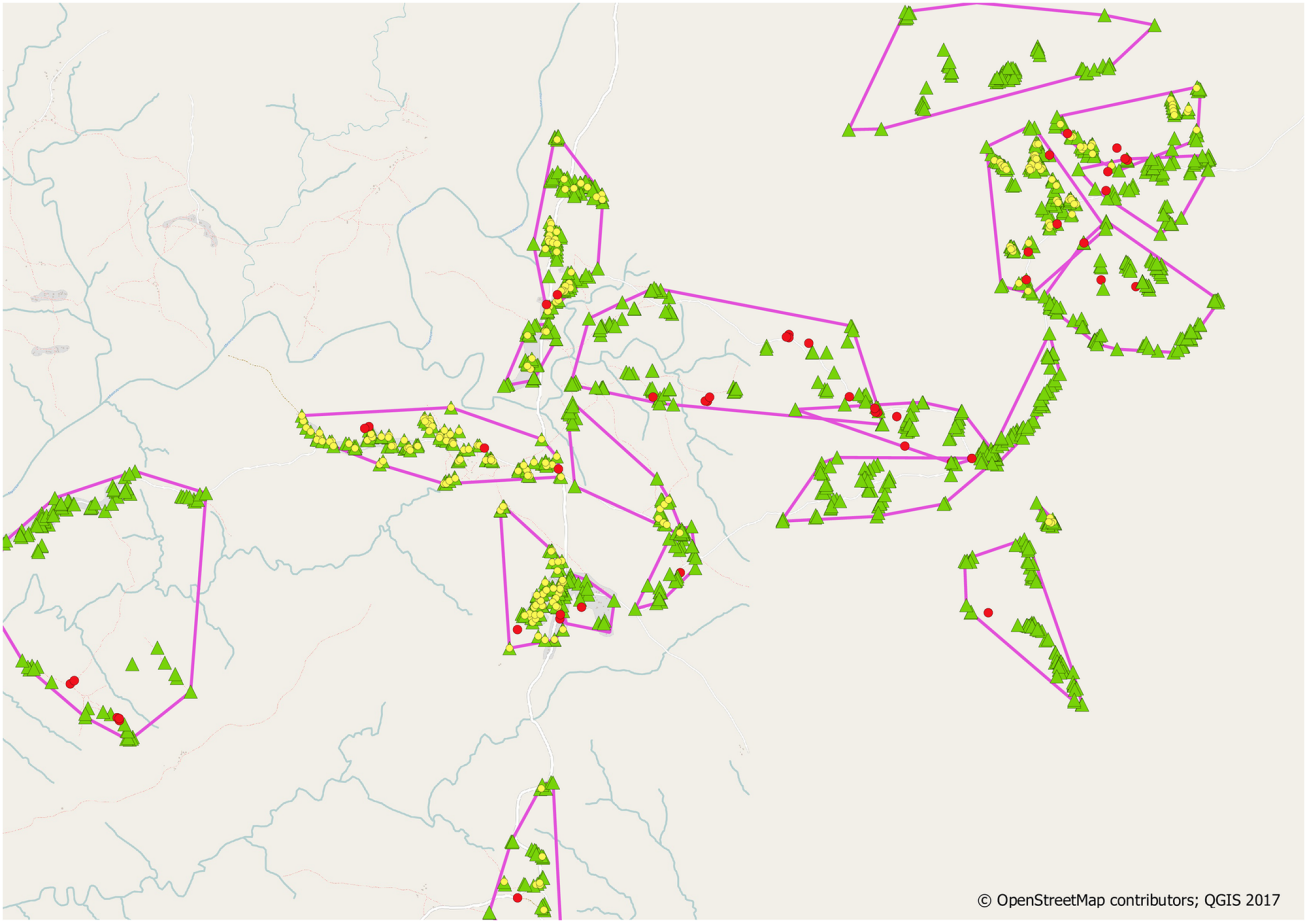
Overlay of crowd-sourced and ground-collected locations. Red pins denote candidate locations for a visit during ground-truthing, i.e. volunteer-provided locations without a GPS-collected match. Green pins are the location recorded as enumerated houses by research assistants during household interviews. Yellow pins are geolocations far from the census one but closer than 40m.

At the end of the census, crowd-sourced geolocations were compared with the GPS-based coordinates collected by the study team to identify locations which were potentially missed in the census. In a first step, data points from the census were processed by grouping the points according to the village in which they were collected using the location id assigned by the fieldworker. A convex hull was placed around the points in each village [27]. Next, the crowd-crafted geolocations were processed sequentially. Points that were located outside a village defined by the convex hull described above were discarded, assuming that these locations were unlikely to be valid locations for the HDSS villages. Points inside a village were classified as either “near” if a geolocation from the census was closer than 40m, or flagged as “distant” if this this was not the case (Fig 4). The rationale for this was that research assistants would visit any house they could see while walking through a village, and we considered it most likely for “distant” locations, if any, to have been missed by the field team’s visual assessments. A random sample of 85 of these locations were mapped and provided to a supervisor for “ground-truthing” to determine the nature of these potential discrepancies, and to estimate the coverage of the population during the census. Fieldworkers, guided by the generated maps and by the coordinates collected for each location, visited the randomized candidate locations and recorded what they observed there. The goal was to verify if these “missing” houses in the census round were meant to be part of the HDSS or for any reason were correctly excluded. Only locations that were identified in all three replicates were eligible for a ground-truthing visit, as the limited time available for visits was focused on the most promising candidate locations.

We also tested if there were houses which were enrolled during the HDSS census but absent from the set of locations identified on satellite images. We used an approach analogous as described above, i.e. we classified HDSS locations as “distant” if they did not have a nearby location among the set of locations identified on the satellite imagery, again using a threshold of 40m.

### Ethical consideration

Ethical clearance for the HDSS was obtained from the University of Malawi, College of Medicine Research Ethics Committee (COMREC) in Malawi (P.05/14/1579). Permissions were obtained from the Ministry of Health and the district health authorities in Chikwawa District. Prior to the start of the study, a series of meetings were held in participating communities to explain the nature and purpose of the study. We obtained individual written informed consent from all participants.

## Results

The census round in the MMP projects started on 20 August 2014 and ended on 14 November 2014, data were collected in one additional village in February 2015. During this census round 6,543 locations and 24,129 individuals were registered in the OpenHDS System.

**Fig 5:**
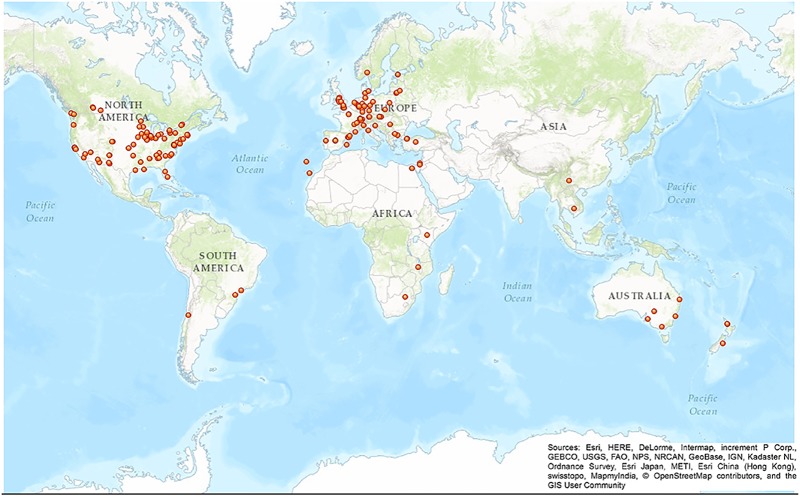
Geographic distribution of volunteers who contributed to the geo-location of buildings.

**Fig 6:**
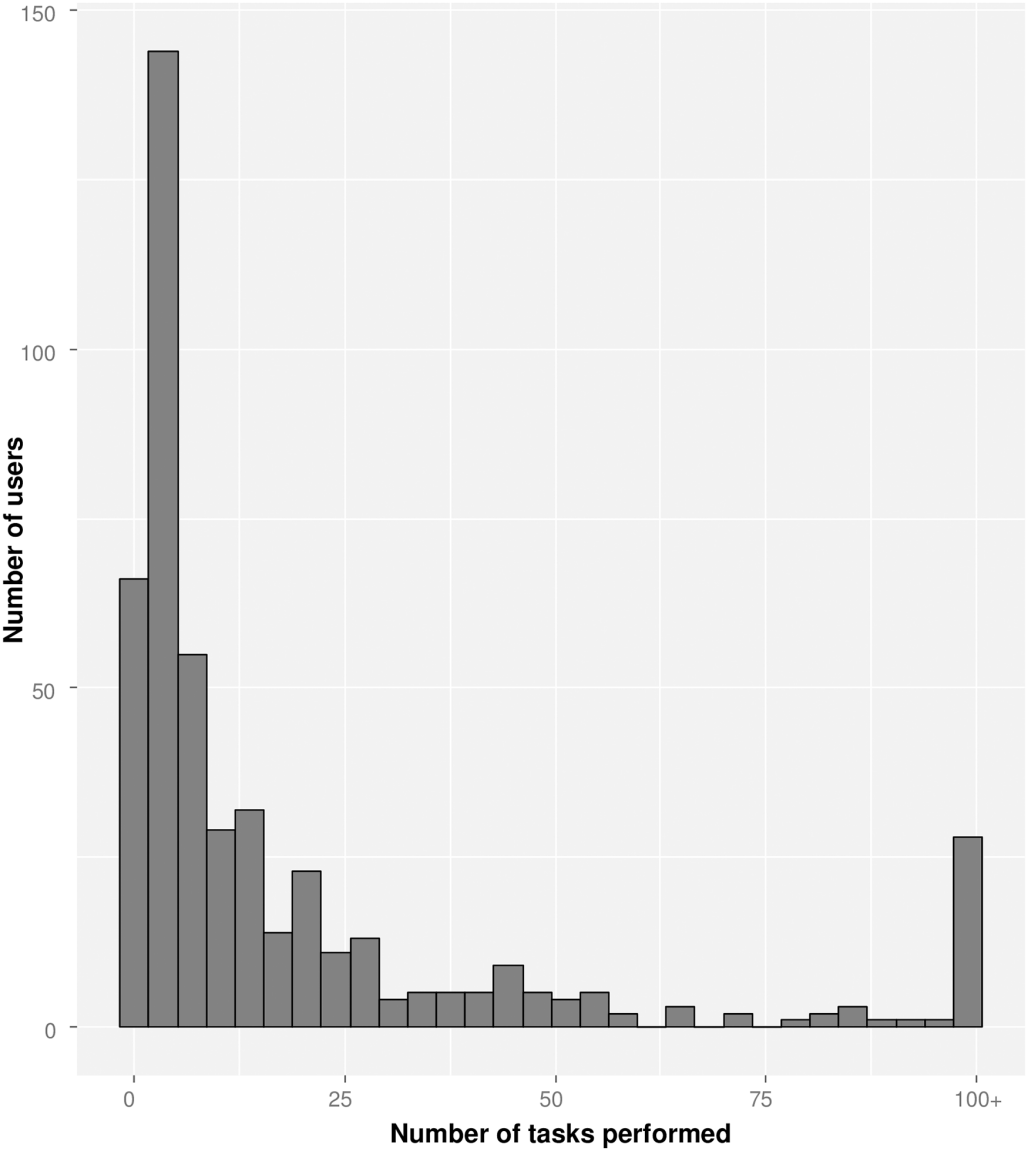
Distribution of numbers of tasks contributed by volunteers. The bin labeled “100+”, contains volunteers who completed 100 or more tasks.

A group of volunteers from more than 30 different countries contributed to the crowd-sourced geolocation effort (Fig 5). 299 registered volunteers (i.e. with a user account on crowdcrafting.org) processed a total of 10,445 task replicates, and unregistered volunteers processed 3,091 task replicates connecting from computers with 174 distinct IP addresses. The processing of the all 4,306 tasks representing the study area was completed within four months. Volunteers contributed a median of 7 task replicates each, but the distribution of task replicates was highly overdispersed, with the top 20 contributors having processed roughly 50% of the tasks (Fig 6).

The data processed and the results of the crowdsourcing are available to be downloaded from the Crowdcrafting.org website [28].

A total of 62,946 geolocations were submitted via mouse-clicks by the volunteers. When applying the replicate-consolidation algorithm to cluster points, a total of 26,247 suspected houses were identified. Of those, 11,046 (42.1%, 95% confidence interval 0.4149 to 0.4268) were confirmed by being identified in all three replicates, and 3,424 of these were within the censused areas (Fig 1). 445 (13.0%, 95% confidence interval 0.1189 to 0.1412) of the volunteer-provided locations within the censused areas were “distant” from the GPS location of the nearest of the 6,543 inhabited houses identified in the HDSS census. Table 1 provides these results disaggregated by focal area. Conversely, 1,490 of the GPS locations of inhabited houses identified in the census were “distant” from the nearest confirmed crowd-sourced location, and 279 censused houses were “distant” from the nearest crowd-sourced location on any of the replicates (Table 2).

**Table 1:** Locations found on satellite imagery.

	Focal Area A	Focal Area B	Focal Area C
**Mouse clicks (across all replicates)**	6749	30235	25962
**Locations identified (through clustering of mouse clicks)**	2769	12780	10698
**Houses (identified in all three replicates)**	1143	5444	4459
**Houses eligible for ground-truthing (as above, but also within a village)**	576	1003	1845
**Houses identified as “distant”**	62	205	178

**Table 2:** Locations found in the HDSS census.

	Focal Area A	Focal Area B	Focal Area C
**Houses enrolled in the HDSS census**	1,157	2,275	3,111
**Houses enrolled in HDSS census which were “distant” compared to the satellite-located houses identified in all three replicates**	320	670	500
**Houses enrolled in HDSS census which were “distant” compared to the satellite-located locations identified in at least one replicate**	122	101	56

In all 85 cases where all three crowd-sourcing replicates identified a house that was absent from the GPS database, ground-truthing indicated that the location had been correctly excluded from census (Table 3). Most of these potential locations vacant or abandoned houses (37 cases, 43.5%) or non-residential buildings such as churches and schools (30 cases, 35.3%).

**Table 3:** Classification of ground-truthed locations: 85 locations were visited after census because the satellite image-sourced locations showed a potentially missed house.

Classification	Number of occurrences	Comments
**Empty House**	37	Uninhabited or abandoned house
**Non-residential building**	30	Schools, churches, health facilities, shops
**Not eligible during census**	6	Constructed or vacant during census
**No building**	6	Tree, anthill, open space
**Other reason**	4	Indistinguishable from census house
**Refused consent**	2	Inhabitants refused consent for participation in the census

For a small number of locations, classification on the ground was not possible. None of these locations were inhabited houses that had been missed during census when empty houses were not taken into account in the system. This indicates that a high percentage of the population coverage was reached using the OpenHDS system in the census round of the HDSS.

A total of 1,490 of the locations visited during the census were found to be distant from all houses identified by volunteers in all three replicates of a task (Table 2). This number was reduced to 279 when considering all locations identified by clustering clicks (irrespective of their presence in other replicates).

## Discussion

The collection of volunteer-provided geolocations for a sizable study area required about the same elapsed time as the ground survey. The crowd-sourcing provided a convincing check of the coverage of the ground census, demonstrating that the HDSS achieved a high coverage of the population of the study area.

However, as implemented, the crowd-sourcing missed many of the inhabited locations.

The cost of crowd-sourcing was negligible because the PyBossa software is a publicly available resource.

The number of houses identified in all three replicates processed by the volunteers, deemed “eligible for ground-truthing” in Table 1 was much lower than the houses enrolled in the HDSS census in the same area. This was both because close-standing buildings cannot always be distinguished on the satellite imagery, and because the algorithm chosen for consolidating replicates groups nearby buildings into single locations.

For the application described here this is of no consequence, except that it introduces an asymmetry between the HDSS and volunteer-provided locations which makes it difficult to compare some results in absolute numbers. For example, the 455 points identified as distant probably represent a higher number of buildings. We think that it would be scientifically interesting to follow up with a more detailed analysis using a supervised learning algorithm [29,30] to explore the potential for locating houses in some of the areas from the volunteer provided data, and then test how it works on the other area(s).

The large number of houses enrolled in the HDSS but not identified in all three replicates of a task was not expected and merits some discussion of possible reasons. One factor that may explain the classification of HDSS-enrolled houses as distant is that for a number of those databases records the reported GPS-accuracy was substantial (up to 50m). A more detailed analysis showed that many of the HDSS houses had close-by analogues in at least one of the task replicates. This raises a number of questions related to the optimal way of presenting tasks to volunteers.

The first concerns the number of replicates. Is three replicates per task are sufficient, or would a higher number of replicates provide a more solid foundation for distinguishing reliably located buildings from spurious mouse clicks? There were 164 tasks in which one replicate was submitted with no clicks, but more than 10 clicks in both other replicates, suggesting that a quorum smaller than the replicate number might increase the quality of volunteered data.

The second is related to task size. It may be useful to make the task size (i.e. the area to be inspected as part of a task) smaller. Due to a glitch of the PyBossa software at the time of the data collection, we lack information on how long it took a volunteer to process each task replicate. This issue has since been fixed, and we recommend that future applications focus on this metric for optimizing the size of the task.

Most of the volunteer work was done by a small number of individuals, whereas most volunteers stopped contributing after a small number of tasks. It is possible that simpler tasks (i.e. smaller area to analyze) would lead to a volunteer contributing more task replicates. Further, it might be possible to identify incentives for those who only contributed a few results to do more, rather than spending effort on recruiting more volunteers [31].

To our knowledge this is the first time that VGI has been employed in an effort to establish the population coverage in an HDSS, and the approach is an important addition to the tools available to HDSS program managers, allowing them to ensure that the entire population was covered during the census or successive rounds. The approach is easily transferable to other areas, and could be used to estimate coverage in any surveillance system which requires geo-locations of houses.

Beyond using the approach described here for quality control in population-based surveillance, we see further applications in the planning of observational or intervention field studies. Potentially, crowd-sourcing of such images could provide improved sampling frames for household surveys, even in areas where there is no population database. This could even be used for generating samples stratified according to other characteristics identifiable on satellite images (e.g. vehicles, or gardens etc.). Similarly, crowd-sourcing could be used to count or localize the numbers of such features within a research area for comparison between different areas. All of these extra studies could be also decided after the data collection and not predetermined a priori.

In general crowdsourcing projects have an outreach component (citizen, civic or amateur science), and the benefit is probably more than the data because people learn about the research [32,33].

VGI [5,34,35] and crowd sourced data (geodata) [36–39] have changed the collection of digital spatial data. This volunteer approach is giving us a new way to improve the data collection, and new ways of comparison.

In last 3–4 years computer image recognition has improved significantly [40], but still this kind of technology is limited to the pharmaceutical or military industry, or in general to research with funding behind and that needs fast response, and analysis of the data, so in general algorithmic image analysis of such remote-sensed images is still challenging [41–43].

Developing and tuning an image analysis application is technically challenging, while crowd sourcing data is relatively straightforward and can be implemented quickly. On the other hand, the problem of identifying houses on satellite images is recurrent and will not go away because even existing population databases need frequent updating, so it is probably worth investing in automating it. VGI, combined with the ground-truthing methodology presented here, may contribute to the process of training image recognition algorithms.
